# Supplementary material for: A Comparative Study of PMETAC-Modified Mesoporous Silica and Titania Thin Films for Molecular Transport Manipulation
Source: Polymers (Basel). 2022 Nov 9;14(22):4823. doi: 10.3390/polym14224823 (PMC9692692; doi:10.3390/polym14224823)
Supplement: Supplementary file 1 [file polymers-14-04823-s001.zip › polymers-1935954-supplementary.pdf]

polymers

MDPI

Supplementary Materials: A Comparative Study of PMETAC-modified Mesoporous Silica and Titania Thin Films for Molecular Transport Manipulation

Sebastian Alberti, Juan Giussi, Omar Azzaroni and Galo J. A. A. Soler-Illia

Table S1. Contact angle of different samples of mesoporous silica (controls) are compared to the full synthesis of the hybrid material. The "X" shows the setps of synthesis done for each sample.

Aptes	Polymer Initiator	monomer	Contact angle
X	X	X	13º
	X		20º
	X	X	30º
		X	15º
X	X		45º
X			25º
			5º

Table S2. Contact angle of different samples of mesoporous titania (controls) are compared to the full synthesis of the hybrid material. The "X" shows the steps of synthesis done for each sample.

Aptes	Polymer Initiator	Monomer	Contact Angle
X	X	X	25º
	X		35º
	X	X	25º
		X	35º
X	X		50º
X			50º
			20º

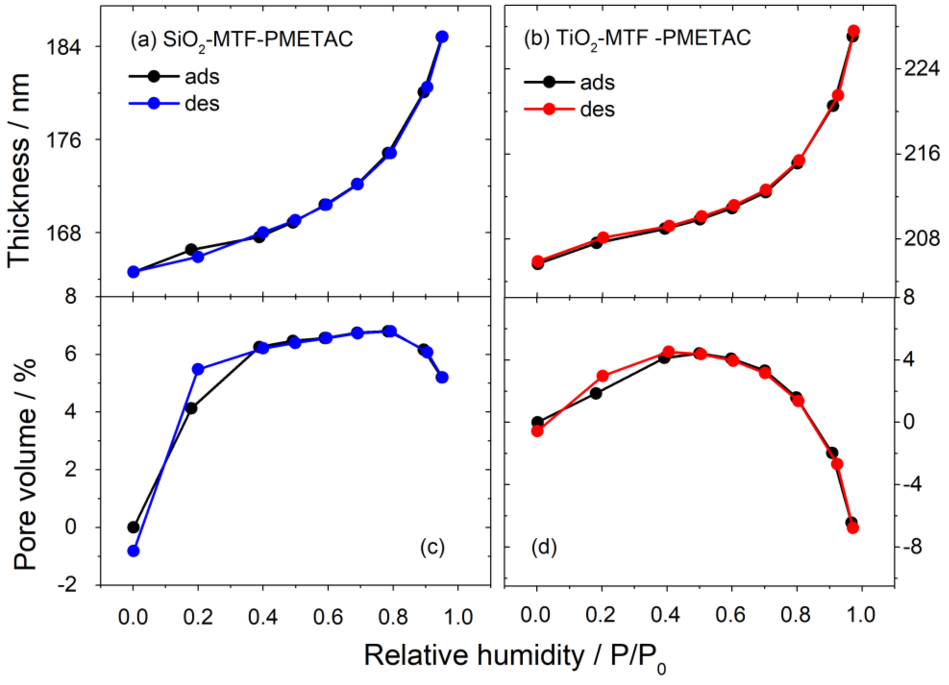

Figure S1. One layer model. Thickness and accessible pore volume of mesoporous thin films after polymerization. PMETAC –silica thickness change upon increments of relative humidity (a) and accessible pore (c) are shown on the left. On the right PMETAC – titania thickness (b) and accessible pore volume (d) is shown. Adsorption and desorption curves are described under the names "ads" and "des" respectively.

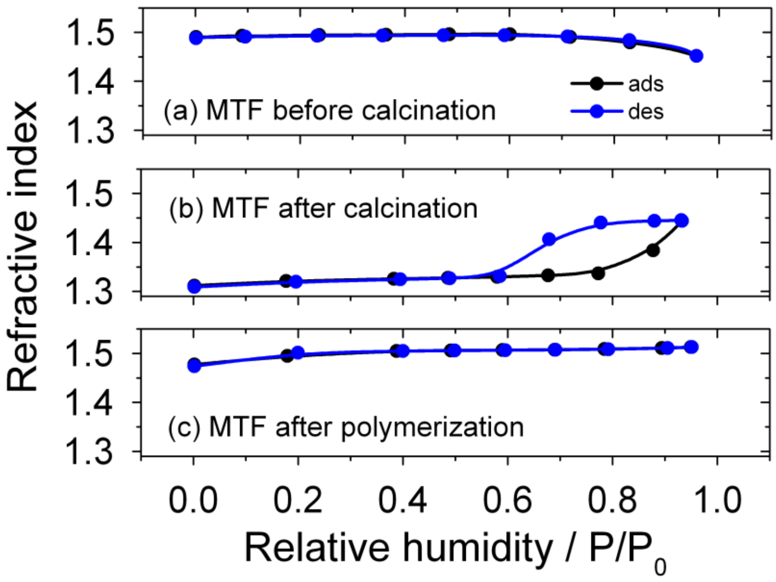

Figure S2. SiO_2-MTF isotherms modification after calcinations and after polymerization. This graphic shows the appearance of hysteresis after calcinations and its disappearance after polymerization. The decrease in the refractive index from 1.5 to 1.3 is equivalent to the increase after polymerization from 1.3 to 1.5. This proves a complete filling of the pores and support the results from the double layer model.
